# Identifying the risk factors associated with food insecurity in the UK veteran population: a nationwide survey

**DOI:** 10.1017/jns.2024.43

**Published:** 2024-10-14

**Authors:** Amy Johnson, Giuseppe Serra, Marco Tomietto, Matthew D. Kiernan

**Affiliations:** 1 The Northern Hub for Veterans’ and Military Families’ Research, Department for Nursing, Midwifery and Health, Northumbria University, Coach Lane Campus, Newcastle upon Tyne, UK; 2 Department of Medicine, Northumbria University Visiting Scholar from The University of Udine, Udine, Italy

**Keywords:** Armed forces, Food insecurity, Health, Military, Veteran

## Abstract

There has been limited focus placed on exploring food insecurity within the UK-ex-Armed Forces population. The present study aims to build on initial work by investigating the prevalence and associated factors of food insecurity within UK veterans and their families and their current health status. 881 veterans (or a family member) who previously served in the Royal Navy and Royal Marines, Army and the Royal Air Force completed an online survey to explore health status, food insecurity and receipt of benefits. In total, 16.9% of survey respondents were part of food-insecure households, with 12% of these also experiencing some element of hunger. Working age, non-officer rank at the time of service discharge, not being married, living in rented accommodation, having at least one medical condition and in receipt of other benefits were significant risk factors associated with food insecurity. Understanding the specific risk factors associated with food insecurity is vital to develop personalised interventions and policies, such as income support programmes and affordable housing initiatives. However, more work is needed to further explore the factors associated with food insecurity, particularly in the long term.

## Introduction

There has been little work focused on understanding the financial situation of UK veterans following discharge from military service.^(1)^ Food insecurity is commonly considered a proxy for financial instability as the quality and amount of food are often forfeited first to afford essential living costs and bills.^(2)^ A recent estimate for UK households experiencing food insecurity was 17%,^(3)^ however, food insecurity appears to be rising. For instance, food banks continue to report an increased unprecedented need for their services and, in some cases, are struggling to fulfil this.^(4–6)^ This rise could be attributed to recent instances of economic instability in the UK, for instance, the COVID-19 pandemic, the cost-of-living crisis, and a repercussion of a declining welfare system.^(4)^

Much of the evidence base of the level of food insecurity experienced by the veteran population has originated from the USA. The prevalence of food insecurity amongst American veterans ranges from 22.5 to 27%,^(7–9)^ with this being lower than non-veterans.^(10,11)^ Key factors related to food insecurity included disability, unemployment, younger age, lower income, being unmarried or not in a relationship, living in households with children, homelessness, ethnicity, and sex.^(7–9,11,12)^ Veterans experiencing food insecurity have reported being unable to afford high-quality food and rely on inexpensive unhealthy food.^(13)^

Food insecurity has been linked to general mental health or psychological distress, depression, diabetes, hypertension, hyperlipidaemia, mobility issues, and cardiovascular disorders in both the general and veteran populations.^(7,8,10,11,14)^ Along with other factors, food insecurity was found to be significantly associated with risk-taking behaviours, such as substance use.^(7)^

Despite sustenance being the second highest identified need reported for Scottish veterans seeking support through Armed Forces charities,^(1)^ research investigating the status of food security within the UK veteran population is scarce. Initial work has shown that one in three veterans, who were receiving support from a service charity, reported low levels of food security and lacked access to affordable food of good quality or quantity.^(15)^ Food insecurity was shown to be significantly higher in veterans who were working age and lived in rented accommodation.^(15)^ It is of note, that this study was completed during the COVID-19 pandemic, which was shown to have impacted food insecurity due to nationwide lockdowns, stock shortages, self-isolation, and loss of income regarding employment in the general population.^(16)^

It is only recently that this work has included veterans who are not in receipt of support estimating that, although 1 in 10 UK veterans were living within a food-insecure household, there was little difference between levels of food insecurity compared to non-veterans.^(17)^ Veterans who were identified as food insecure were more likely to be younger and in receipt of disability benefits.^(17)^ Whilst these initial studies are valuable, the research completed in the USA suggests that food insecurity could be a significant issue to the ex-Armed Forces Community. Presently there has been limited focus on the impact of food insecurity experienced by the UK-ex-Armed Forces population. Therefore, further evidence is required not only to understand the situation of food insecurity among UK veterans but to also identify risk factors to inform service provision and planning to meet need of individuals before they present in crisis.

This study aims to explore the status of food insecurity within the UK-ex-Armed Forces population, who may or may not be receiving support from a service charity. The aims of this study were twofold; to explore the levels of food security and health status of UK veterans and their families, and to identify the variables associated with food insecurity in the UK veteran population.

## Methods

### Study design

This study adopted a cross-sectional design to identify instances of food insecurity through online surveys administered to UK Royal Navy (RN), Royal Marines (RM), Army, and Royal Air Force (RAF) veterans and their families. The online surveys were hosted via Joint Information Systems Committee (JISC) online surveys and were available between 1^st^ February and 31^st^ March 2023.

Male and female UK veterans and their families across all military services were invited to complete the online survey. This was a convenience sample with recruitment being supported by The Royal Naval Association, The Royal Marine Charity, and the RAF Association who disseminated information about the study and the survey link to their members across the UK (England, Scotland, Wales and Northern Ireland). Participating in the survey did not impact membership to the service association or any ongoing support being received. As there is currently no singular charity or association for the Army, a key point of contact through the Ministry of Defence disseminated the online survey link to Corps and Regimental Association members. The online surveys were promoted through Twitter, Facebook, and LinkedIn to capture the experiences of veterans not necessarily associated with their service charity or association.

### Ethical approval

This study was conducted according to the guidelines laid down in the Declaration of Helsinki and all procedures involving human subjects/patients were approved by the Northumbria University Health and Life Sciences Ethics Committee (Reference Number: 1628). Written informed consent was obtained from all subjects/patients. The Strengthening the Reporting of Observational Studies in Epidemiology (STROBE) guidelines were adopted.

### Participants

There was a total of 908 responses to the online survey. Twenty-seven responses were removed due to declining to complete the survey (n = 18), uncompleted responses (n = 2), still serving at the time of survey completion, and not being UK veterans or family member (n = 7). In total, 881 responses were included in the analysis. Table 1 presents participant characteristics. For ethnicity and gender, categories containing under 5 participants were removed to protect confidentiality and reduce the risk of breach of privacy.^(18)^

**Table 1.** Survey respondents characteristics (N = 881)^*^

Variables	Total number and percentage
Age (n = 862)	Mean = 66.58 (SD = 10.75)
Non-working age (over 66 years)	496 (57.5%)
Working age (under 66 years)	366 (42.5%)
Gender (n = 868)	
Male	727 (83.9%)
Female	140 (16.1%)
Service (N = 881)	
Army	443 (50.3%)
Royal air force	142 (16.1%)
Royal navy and royal marines	296 (33.6%)
Military background (n = 870)^*^	
Served in the regulars	785 (90.2%)
Served in the reserves	38 (4.4%)
Family member served in the regular forces	29 (3.3%)
Family member served in the reserves forces	2 (0.2%)
Other	16 (1.8%)
Military rank (n = 811)	
Officer	176 (21.9%)
Non-officer	626 (78.1%)
Senior officer	125 (15.6%)
Junior officer	51 (6.4%)
Senior NCO	326 (40.6%)
Junior non-commissioned officer	207 (25.8%)
Junior rank rate	93 (11.6%)
Length of service (in years) (n = 797)	Mean = 18.99 (SD = 10.85)
Years since discharge (n = 790)	Mean = 30.00 (SD = 15.36)
Ethnicity (n = 879)	
White	864 (98.3%)
Other	15 (1.7%)
Marital status (n = 881)	
Married/co-habiting	645 (74.6%)
Single	46 (5.3%)
Separated/divorced	83 (9.6%)
Widowed	91 (10.5%)

* Groups containing below 5 cases were removed to preserve participant identity and as a privacy risk in accordance with guidance from the Information Commissioner’s Office.^(18)^

Considering a confidence level of 95% and a power level of 80%, the needed sample size to determine a minimum OR of 1.3 was 721 participants.^(19)^

### Data collection

*Food Insecurity:* Ten items from The United States Department of Agriculture (USDA) Food Insecurity Scale were utilised to assess instances of household food insecurity over the last 30 d.^(20)^ This scale has been widely validated,^(21)^ and it is currently the one used by the department of work and pension to measure food insecurity among UK general population.^(22)^ The same scale was used in a pilot study on food insecurity among UK veterans conducted in 2021.^(15)^

Affirmative responses were summed to provide an overall score and to provide a category as to the level of household food insecurity. This score is allocated to one of four categories: ‘Food secure’, ‘Food Insecure Without Hunger’, ‘Food insecure with Hunger (Moderate)’, and ‘Food Insecure with Hunger (Severe)’. These categories were further condensed as ‘Food Secure’ and ‘Food Insecure’ and an additional binary variable was developed indicating whether hunger was established for analysis. The Cronbach’s Alpha for the USDA was calculated as .933 for the dataset in this study, indicating a high level of internal consistency.

*Mental Wellbeing:* The Short Warwick-Edinburgh Mental Wellbeing Scale (SWEMWBS)^(23,24)^ assessed levels of mental wellbeing in survey respondents. SWEMWBS consists of 7 questions rated on a 5-point Likert scale, ranging from ‘None of the time’ to ‘All of the time’. Initial total scores were then transformed into metric scores in accordance with SWEMWBS guidance. The final scores range between 7 and 35, with a higher score indicating a higher level of mental wellbeing, and can be compared to the mean population mental wellbeing score of 23.5.^(25)^ The internal consistency for SWEMWBS was high as indicated by a score of .907 for Cronbach’s Alpha in this study.

*Health:* Survey respondents were asked to self-report their general health status on a 5-point Likert scale ranging from ‘very good’ to ‘very poor’. For the regression analysis, ‘very good’ and ‘good’ were aggregated as ‘good health’ while ‘poor’ and ‘very poor’ were aggregated as ‘poor health’. The intermediate value was considered missing, as the ‘poor health’ group was compared to the ‘good health’ group. Participants also reported any long-term conditions that were not compensated under the Armed Forces Compensation Scheme and/or the War Pension Scheme.^(25)^ This was assessed as a binary variable; i.e. those who reported experiencing or not experiencing a long-term condition.

*Military Demographics:* All survey respondents were asked to provide their prior military background, categorised as having previously served in the Regular service, previously served in the Reserves, a family member of an individual who served in the Regular service, or a family member of an individual who served in the Reserves. Participants who had previously served in the military provided the length of service and rank and years since discharge. Ranks were categorised as both binary (i.e. ‘Officer’ and ‘Non-Officer’) and categorical variables (‘Junior Rank Rate’, ‘Junior Non-Commissioned Officer (NCO)’, ‘Senior NCO’, ‘Junior Officer’, and ‘Senior Officer’). A further summary of the rank categorical variable is provided in Table 2 (see Supplementary Material).

*Participant Demographics:* Participant demographics explored: gender (‘male’ or ‘female’), ethnicity (‘White’ and ‘Other’), present living situation (i.e. living alone or living with others), and age (‘working age’ for veterans aged under 66 and ‘non-working age’ for veterans aged 66 and over). The choice of dividing the veterans into two age groups is based on previous literature, as being in working age was found to be a risk factor for poor social outcomes.^(1)^ However, the age at which each individual retires varies depending on multiple factors, so there is no unique cut off. For the purpose of this paper, the age of 66 was defined as the age of retirement, according to the active legislation at the moment of the data collection.

Additional variables included marital status (‘Single’, ‘Married/Co-habiting’, ‘Separated/Divorced’ and ‘Widowed’), housing status (‘Owner Occupied’, ‘Rented’, ‘Comes with Occupation’, ‘Other’) and employment status (‘Employed’, ‘Unemployed’, ‘Retired’, and ‘Other’). These variables were further transformed as follows for the regression analysis: marital status (‘Married/Co-habiting’ and ‘Single/Separated/Divorced/Widowed’), housing status (‘Owner’ and ‘Rented’; the value ‘Comes with Occupation’ and ‘Other’ were considered missing values), and employment status (‘Employed’, ‘Unemployed’, ‘Retired’; ‘Other’ was considered missing value).

*Financial Benefits:* Financial benefits were categorised as binary variables, i.e., those in receipt or not receiving support through The Armed Forces Compensation Scheme, The War Pension Scheme, a service charity and the Department of Work and Pensions.

### Data analysis

STATA®17^(26)^ was used to analyse continuous and categorical variables, and confidence intervals. Continuous variables were described as means and standard deviations before groups were assessed for statistical differences using a two-sample *t*-test. Categorical variables are presented as frequency and percentages. Group differences were assessed using chi-square test,^(27)^ with the Fisher exact test being used for cases below 5. For larger tables with low counts, the Fisher test was conducted with the Freeman-Halton extension.

The logistic regression analysis^(28)^ was conducted using STATA®17.^(26)^ Initially, a univariable logistic regression was performed to obtain Odds Ratios and confidence intervals for each factor. To investigate potential causal pathways leading to the outcome, a Direct Acyclic Graph (DAG) was constructed to identify possible confounders and mitigate the risk of spurious correlations. DAGs are a well-established tool used by researchers to identify sources of bias when addressing specific causal research questions.^(29)^ A multivariable logistic regression was then performed with all the variables found to be significant in the univariable models. Two covariates were removed from the model due to multicollinearity, namely ‘living alone’ (strongly collinear with marital status) and ‘employment’ (strongly collinear with age). Covariates considered potential a priori confounders (gender and service) were retained even if not significant in the univariable model. The reference categories were chosen to highlight a higher risk rather than a protective effect of some factors.

As a sensitivity analysis, different cutoffs and categorizations were explored for variable dichotomisation, including the division of variables into more than two categories. However, these alternative approaches did not yield significant changes in the results (supplementary file). All analyses were performed with a confidence level of 95%. Any missing data was addressed using listwise deletion.

## Results

In total, 16.9% of veterans and their families were found to be part of food-insecure households (95% CI = 15.6–18.2), with 12% experiencing some element of hunger (95% CI = 10.9–13.1). Of note, 37.9% of participants self-reported their health as good (95% CI = 36.3–39.5), and the average wellbeing level was 23.4 (95% CI = 23.05–23.72). The proportion of the sample in receipt of financial benefits was small, particularly recipients of financial support due to injury obtained during or due to service; 6.5% receiving The Armed Forces Compensation Scheme (95% CI = 5.6–7.3), and 19.1% receiving a War Pension (95% CI = 17.7–20.4). Table 3 presents the descriptive statistics and confidence intervals.

**Table 3.** Characteristics of the sample

Variables	Total number and percentage	Confidence interval (95%)
Food secure (N = 881)	732 (83.1%)	81.8–84.4
Food insecure (N = 881)	149 (16.9%)	15.6–18.2
Food insecure with hunger (N = 881)	106 (12%)	10.9–13.1
Level of mental wellbeing (n = 857)	Mean = 23.38	23.1–23.7
Reported health status (n = 876)		
Very good	140 (16%)	14.7–17.2
Good	332 (37.9%)	36.3–39.5
Fair	262 (29.9%)	28.4–31.5
Poor	98 (11.2%)	10.1–12.3
Very Poor	44 (5%)	4.3–5.8
Sufferers of long-standing medical conditions (not compensated under armed forces compensation scheme and/or the war pension scheme) (n = 845)	435 (51.5%)	49.8–53.2
Housing situation (n = 877)		
Owner occupied (mortgage or owned outright)	697 (79.5%)	78.1–80.8
Rented (housing association, private landlord or local authority)	155 (17.7%)	16.4–19
Comes with occupation (or service family home)	8 (0.9%)	0.6–1.2
Other	17 (1.9%)	1.5–2.4
Living alone (n = 845)		18.6–21.4
Employment status (n = 878)		
Retired	494 (56.3%)	54.6–57.9
Employed	285 (32.5%)	30.9–34
Unemployed	16 (1.8%)	1.4–2.3
Other	83 (9.5%)	8.5–10.4
In receipt of benefits		
Armed forces compensation scheme (n = 864)	56 (6.5%)	5.6–7.3
War pension (n = 865)	165 (19.1%)	17.7–20.4
Financial assistance from service charity (n = 879)	28 (3.2%)	2.6–3.8
Benefits from the department of work and pensions (n = 869)	218 (25.1%)	23.6–26.6

### Cross group comparison

Survey respondents who were food insecure were younger compared to those who were food secure (60.86 ± 10.75 vs 67.75 ± 10.38, P < 0.001) (see Table 4). Of those reporting instances of food insecurity, working-age participants had statistically higher levels compared to their older non-working-age counterparts, (25.1% vs 10.9%, P < 0.001). Instances of food insecurity were greater in participants who were single and separated/divorced compared to those who were married/co-habiting or widowed (respectively 43.5% and 32.5% vs 12.4% and 19.8%, P < 0.001). Those in rented accommodation or reported ‘other’ reported higher levels of food insecurity compared to owner occupied and in accommodation that was provided with occupation (respectively 41.9% and 47.1% vs 10.5% and 25.0%, P < 0.001). Similarly, those who were unemployed or reported ‘other’ had higher levels of food insecurity compared to those employed and retired (respectively 43.8% and 44.6% vs 16.8% and 11.5%, P < 0.001).

**Table 4.** Characteristics of the level of food security

Variable	Food secure	Food insecure	T value/ chi-square	P value
Age (n = 862)	Mean = 67.75 (SD = 10.38)	Mean = 60.86 (SD = 10.75)	7.273	<0.001
Non-working age	442 (89.1%)	54 (10.9%)	30.395	<0.001
Working age	274 (74.9%)	92 (25.1%)		
Gender (n = 867)				
Male	611 (84.0%)	116 (16.0%)	1.908	0.167
Female	111 (79.3%)	29 (20.7%)		
Service (N = 881)				
Army (n = 443)	362 (81.7%)	81 (18.3%)	4.875	0.087
Royal air force (n = 142)	127 (89.4%)	15 (10.6%)		
Royal navy and royal marines (n = 296)	243 (82.1%)	53 (17.9%)		
Military background (n = 870)				
Served in the regulars	655 (83.4%)	130 (16.6%)	1.495	0.813
Served in the reserves	30 (78.9%)	8 (21.1%)		
Family member served in the regulars	23 (79.3%)	6 (20.7%)		
Family member served in the reserves	2 (100.0%)	0 (0.0%)		
Other	13 (81.3%)	3 (18.8%)		
Military rank (n = 811)				
Officer	169 (96.0%)	7 (4.0%)	26.26	<0.001
Non-officer	499 (79.7%)	127 (20.3%)		
Senior officer	122 (97.6%)	3 (2.4%)	76.468	<0.001
Junior officer	47 (92.2%)	4 (7.8%)		
Senior NCO	290 (89.0%)	36 (11.0%)		
Junior non-commissioned officer	153 (73.9%)	54 (26.1%)		
Junior rank rate	56 (60.2%)	37 (39.8%)		
Length of service (in years) (n = 797)	Mean = 20.07 (SD = 10.84)	Mean = 13.53 (SD = 9.18)	6.481	<0.001
Years since discharge (n = 790)	Mean = 29.87 (SD = 15.65)	Mean = 30.67 (SD = 13.87)	–0.54	0.589
Ethnicity (n = 879)				
White	720 (83.3%)	144 (16.7%)	0.118	0.726
Other	12 (80.0%)	3 (20.0%)		
Marital status (n = 881)				
Married/co-habiting	565 (87.6%)	80 (12.4%)	47.698	<0.001
Single	26 (56.6%)	20 (43.5%)		
Separated/divorced	56 (67.5%)	27 (32.5%)		
Widowed	73 (80.2%)	18 (19.8%)		
Mental wellbeing (n = 857)	Mean = 24.48 (SD = 4.51)	Mean = 17.86 (SD = 3.15)	16.741	<0.001
Reported health status (n = 876)				
Very good	136 (97.1%)	4 (2.9%)	149.744	<0.001
Good	310 (93.4%)	22 (6.6%)		
Fair	207 (79.0%)	55 (21.0%)		
Poor	56 (57.1%)	42 (42.9%)		
Very poor	18 (40.9%)	26 (59.1%)		
Non-sufferers of long-standing medical Conditions (not compensated) (n = 410)	370 (90.2%)	40 (9.8%)	29.098	<0.001
Sufferers of long-standing medical conditions (not compensated) (n = 435)	332 (76.3%)	103 (23.7%)		
Housing situation (n = 877)				
Owner occupied (mortgage or owned outright)	624 (89.5%)	73 (10.5%)	86.468	<0.001
Rented (housing association, private landlord or local authority)	90 (58.1%)	65 (41.9%)		
Comes with occupation (or service family home)	6 (75.0%)	2 (25.0%)		
Other	9 (52.9%)	8 (47.1%)		
Living with others (n = 676)	573 (84.8%)	103 (15.2%)	3.619	0.057
Living alone (n = 169)	133 (78.7%)	36 (21.3%)		
Employment status (n = 878)				
Retired	437 (88.5%)	57 (11.5%)	63.389	<0.001
Employed	237 (83.2%)	48 (16.8%)		
Unemployed	9 (56.3%)	7 (43.8%)		
Other	46 (55.4%)	37 (44.6%)		
Benefits				
In receipt of armed forces compensation scheme (n = 56)	40 (71.4%)	16 (28.6%)	5.811	0.016
Not in receipt of armed forces compensation scheme (n = 808)	678 (83.9%)	130 (16.1%)		
In receipt of war pension (n = 165)	130 (78.8%)	35 (21.2%)	2.893	0.089
Not in receipt of war pension (n = 700)	590 (84.3%)	110 (15.7%)		
Received financial assistance from service charity (n = 28)	10 (35.7%)	18 (64.3%)	46.501	<0.001
Did not receive financial assistance from service charity (n = 851)	721 (84.7%)	130 (15.3%)		
Received benefits from the Department of Work and Pensions (n = 218)	140 (64.2%)	78 (35.8%)	74.987	<0.001
Did not receive benefits from the Department of Work and Pensions (n = 651)	583 (89.6%)	68 (10.4%)		

In terms of health, food insecurity was statistically related to lower mental wellbeing (17.86 ± 3.15 vs 24.48 ± 4.51, P < 0.001). Of those reporting food insecurity, higher levels were found in those reporting poor or very poor health, compared to survey respondents reporting very good, good, or fair health (respectively 42.9% and 59.1% vs 2.9%, 6.6% and 21.0%). Survey respondents who reported having a long-standing medical condition (not receiving compensation under the Armed Forces Compensation Scheme and/or the War Pension Scheme) had higher instances of food insecurity (23.7% vs 9.8%, P < 0.001).

Compared to those not in receipt of financial support, a higher level of food insecurity was found in respondents who received support through The Armed Forces Compensation Scheme (28.6% vs 16.1%, P < 0.016), a service charity (64.3% vs 15.3% P < 0.001), and the Department of Work and Pensions (35.8% vs 10.4%, P < 0.001).

Regarding factors specific to veteran participants, non-officers rated higher instances of food insecurity (20.3% vs 4.0%, P < 0.001), an effect that decreased with higher ranks. Veterans who reported a junior rank rate, junior and senior NCOs had higher instances of food insecurity compared to junior and senior officers (respectively 39.8%, 26.1% and 11.0% vs 7.8% and 2.4%, P < 0.001). Finally, veterans who served for a shorter length of time reported statistically lower levels of food insecurity (20.07 ± 10.84 vs 13.53 ± 9.18, P < 0.001).

### Regression model

In the univariable model (see Table 5), working age was found to have a significant association with food insecurity (OR = 2.75, 95% CI = 1.9–3.97, P < 0.001). Similarly, non-officer rank (OR = 4.9, 95% CI = 2.23–10.8, P < 0.001), not being married (OR = 2.96, 95% CI = 2.04–4.3, P < 0.001), having at least one medical condition (OR = 2.87, 95% CI = 1.94–4.26, P < 0.001), living in a rented housing (OR = 6.17, 95% CI = 4.14–9.22, P < 0.001), and receiving other benefits (OR = 2.06, 95% CI = 1.39–3.05, P < 0.001) were all significantly associated with an increased risk of food insecurity.

**Table 5.** Logistic regression identifying factors associated with food insecurity

	Univariable model			Multivariable model		
Risk factor	Odds ratio	95% CI	P	Odds ratio	95% CI	P
	Age			Age		
Non-working age	1			1		
Working age	2.75	1.9–3.97	<0.001	5.93	3.08–11.41	<0.001
	Gender			Gender		
Male	1			1		
Female	1.38	0.87–2.17	0.168	1.2	0.55–2.65	0.648
	Service			Service		
Army	1			1		
RAF	0.53	0.29–0.95	0.033	0.91	0.39–2.12	0.835
Royal navy	0.97	0.66–1.43	0.896	1.53	0.79–2.97	0.206
	Rank			Rank		
Officer	1			1		
Non-officer	4.9	2.23–10.8	<0.001	4.29	1.62–11.37	0.003
	Marital status			Marital status		
Married	1			1		
Not married	2.96	2.04–4.3	<0.001	3.36	1.74–6.5	<0.001
	Chronic conditions			Chronic conditions		
No medical condition	1			1		
At least 1 medical condition	2.87	1.94–4.26	<0.001	3.93	2.1–7.34	<0.001
	Housing			Housing		
House owner	1			1		
Rented accommodation	6.17	4.14–9.22	<0.001	3.5	1.84–6.67	<0.001
	Living alone			Living alone		
Living with others	1			1		
Living alone	1.51	0.99–2.3	0.058			
	Employment			Employment		
Retired	1			1		
Employed	1.55	1.03–2.35	0.038			
Unemployed	5.96	2.14–16.63	0.001			
	Other benefits			Other benefits		
Does not receive benefits	1			1		
Receives benefits	2.06	1.39–3.05	<0.001	2.19	1.2–4.01	0.011

After adjusting for other variables in the multivariable model, working age remained significantly associated with food insecurity (OR = 5.93, 95% CI = 3.08–11.41, P < 0.001). Additionally, non-officer rank (OR = 4.29, 95% CI = 1.62–11.37, P = 0.003), not being married (OR = 3.36, 95% CI = 1.74–6.5, P < 0.001), having at least one medical condition (OR = 3.93, 95% CI = 2.1–7.34, P < 0.001), living in a rented housing (OR = 3.5, 95% CI = 1.84–6.67, P < 0.001), and receiving other benefits (OR = 2.19, 95% CI = 1.2–4.01, P = 0.011) remained significantly associated with an increased risk of food insecurity. The proportion of variance explained by the model resulted to be 28.2%.

Gender and service did not show significant associations with food insecurity in either the univariable or multivariable models.

## Discussion

This study aimed to explore the prevalence of food insecurity and health status experienced by UK veterans and their families, and to identify key variables associated with food insecurity. The study found that 16.9% of survey recipients lived in food-insecure households with 12% experiencing hunger. This percentage reflects recent estimates for UK-wide households experiencing food insecurity^(3)^ but is lower than the estimated prevalence in USA veterans,^(7–9)^ which could highlight key international differences. Regarding UK veterans, the percentage of food insecurity appears to be higher than that of previous research undertaken on the UK veteran population^(17)^ but lower than the one recorded in veterans accessing charities.^(15)^ Whilst the present percentages do indicate that some veterans experience instances of food insecurity, and in some cases hunger, this appears to reflect the prevalence within the general population^(3)^ as opposed to highlighting a veteran-specific need.

The cross-group comparison via chi-square tests highlighted that younger, working-age participants were more likely to report higher levels of food insecurity compared to their older counterparts. These findings support previous work assessing food insecurity within the UK^(15,17)^ and USA^(8,11,12)^ veteran population. Possible explanations could include that older non-working-age participants are less likely to be living with dependents (i.e. children) and could be in a stronger financial and living situation, such as owning their own house. Specific to the military population, service leavers are eligible to receive the Armed Forces Pension Scheme (AFPS) which varies depending on their rank at the time of discharge. There are presently three possible armed forces pension schemes that a veteran might be in receipt of or eligible for; 1975, 2005 and 2015.^(30–36)^ With the mean age of the participants in the sample being 66.58 years old, it is possible that the older participants who completed this survey were recipients of the 1975 AFPS. In this case, veterans were entitled to receive their pension immediately following retirement if they were aged under 55 years, provided they had served the minimum length of service for their rank (16 years as an officer and 22 years for other ranks).^(30,34)^ This minimum year of service was removed in subsequent pension schemes which stated that pension was only available following the age of 55^(29,33)^ or 60.^(30,31)^ Provided their age and choice, this could allow veterans retiring under AFPS 1975 terms the ability to continue working during their civilian life whilst still receiving military benefits.

Similarly, survey respondents who were single or separated/divorced, living in rented accommodation, unemployed or receiving support from the Department of Work and Pensions were more likely to be food insecure. Again, these findings support that of previous literature.^(9,12,15,17)^ These factors could be directly related to financial status. For instance, individuals who are single or divorced are more likely to be living in a single-income household and those unemployed are likely to be reliant on savings or income support. Due to these factors, it is possible that both quality and amount of food consumed is impacted to prioritise other essential bills.^(2)^

The findings of this study noted the prevalence of lower mental wellbeing, poor or very poor health and reported long-standing medical condition in those with higher levels of food insecurity. Previous literature has found associations between food insecurity and veteran’s general and physical health,^(7,8)^ mental health,^(7,8,11)^ and long-term conditions and their management, such as diabetes.^(7)^ Future research could further explore the association between food insecurity and health, particularly within the veteran population where it is possible that long-standing conditions due to their military service could entitle them to further compensation through The War Pension Scheme and the Armed Forces Compensation Scheme.^(25)^ Both funding streams also supports bereaved military spouses if their partner’s death was linked to their military service. The present study did explore these factors, however, only the Armed Forces Compensation Scheme (AFCS) had a trend towards statistical significance. In terms of injury or illness, since initial conception, 107,333 claims have been cleared under the AFCS of which 61,819 were accepted and received a form of financial support, either lump sum or a life-long Guaranteed Income Payment.^(34)^ As only 6.4% of the present sample reported receiving this as a form of support, exploring this possible correlation with a larger sample size could provide more insight.

This is the first study to explore the impact of rank at time of military discharge on the prevalence of food insecurity. The cross-group comparison identified that veterans who were not officers at time of discharge were more likely to report instances of food insecurity and this was identified as a key variable within the regression model. The rank at time of discharge was used as a proxy measurement for educational status and received pension benefits. While it is true that rank does not always accurately reflect educational status, there are some minimum education requirements for officers; in addition, a proportion of the officers is professionally qualified (medics, engineers etc.).^(37)^ Based on this, we assume that, on average, officers possess a higher educational status than non-officers.

All available armed forces pensions determine financial entitlement based on rank at the time of leaving service,^(30–34)^ with officers being more likely to receive higher payments due to their previous salary. Similarly, under the 1975 AFPS, officers were able to retire earlier from service which could provide more opportunities for a career change. It is possible that this financial stability would impact positively on levels of food security.

The regression model identified key variables associated with food insecurity, specifically working age, non-officer rank, not being married, having a long-term medical condition, living in rented accommodation, and receiving other benefits. This is valuable information in developing targeted interventions, for instance affordable housing initiatives, and healthcare interventions targeted directly to those more at risk. Moreover, these results contribute to identifying a profile of the veterans at risk of food insecurity, to proactively plan health and social care interventions to prevent food insecurity itself. This has the potential to enable a more effective allocation of support and resources and to improve the individual mental and physical wellbeing of this population over time. Future research could include longitudinal studies to fully explore the relationship between these risk factors and food insecurity. Additionally, incorporating qualitative elements could provide a deeper understanding of the impact of food insecurity and identify additional risk factors for investigation based on veteran’s views.

The key strengths of this study include the large sample size, which is more representative than previous research exploring the prevalence of food insecurity within the UK-ex-Armed Forces population, allowing to detect an odds ratio of 1.3 with a power of 80%.

This is also the first study to explore the influence of different services and rank upon leaving the service on instances of food insecurity. Whilst the sample size of 881 participants was over the necessary minimum of 721 for a power level of 80%,^(19)^ the representation from RAF veterans (n = 142) was much lower than the other services (Royal Navy & Royal Marines (RNRM) = 296 and Army = 443). Future work could further explore the impact of service on instances of food insecurity with a more equal sample size to fully capture any further associated variables.

The present study relied on self-reported responses and this research approach has several limitations. For example, the subjective interpretation or misunderstanding of some questions could bias some of the responses. Whilst these responses were rectified where possible, and removed if unclear, this is a limitation to consider. Furthermore, self-reported survey might be biased by a social desirability bias that could lead to an under- or over-estimation of some responses based on the conformity of the answer to societal norms. Despite these points, it is important to note that both the USDA and SWEMWBS had high levels of internal consistency with Cronbach’s Alpha levels of over 0.90. This indicates that the measure of food insecurity and mental wellbeing are reliable in the current sample.

An additional consideration is that the sample was recruited via email, and this could have introduced some level of bias as the older generation may be less likely to access an online survey. However, 57.5% of the participants in this study were aged over 66, a percentage comparable to that of the general veteran population.^(38)^ The online survey may have also pre-selected a sample of veterans with higher incomes, as the poorest subjects may be less likely to have an internet connection or own computer. This means that the overall prevalence of food insecurity could be even higher than the one recorded in this study. Moreover, the cross-sectional design did not allow to identify possible changes on the prevalence of food insecurity before and after major societal events, such as the COVID-19 pandemic.

Overall, these results highlight the multifaceted nature of food insecurity in the ex-Armed Forces population, and its complex interplay with various socio-demographic factors. Understanding the causal pathways and mechanisms by which these risk factors contribute to food insecurity is crucial for developing targeted interventions and policies to address this issue. Efforts should be directed toward income support programmes, affordable housing initiatives, and healthcare interventions targeted directly to those that were found to be more at risk. Additionally, longitudinal studies are needed to investigate the temporal relationships between these risk factors and food insecurity, further explaining the underlying dynamics and facilitating more effective preventive strategies.

## Supporting information

Johnson et al. supplementary material Johnson et al. supplementary material

## Supplementary material

The supplementary material for this article can be found at https://doi.org/10.1017/jns.2024.43

## Abbreviations

**AFPS:** Armed Forces Pension Scheme; **CI:** Confidence Interval; **DAG:** Direct Acyclic Graph; **NCO:** Non-Commissioned Officer; **OR:** Odds Ratio; **RAF:** Royal Air Forces; **RM:** Royal Marines; **RN:** Royal Navy; **STROBE:** Strengthening the Reporting of Observational Studies in Epidemiology; **SWEMWBS:** Short Warwick-Edinburgh Mental Wellbeing Scale; **USDA:** United States Department of Agriculture.
