# Supplementary material for: Identifying the risk factors associated with food insecurity in the UK veteran population: a nationwide survey
Source: J Nutr Sci. 2024 Oct 14;13:e63. doi: 10.1017/jns.2024.43 (PMC11503827; doi:10.1017/jns.2024.43)
Supplement: Johnson et al. supplementary material [file S2048679024000430sup001.docx]

# **Supplementary Material**

Military ranks were categorised according to guidance regarding the Armed Forces Pension Scheme (35). By using this measure, a veteran’s rank at the time of leaving service provided a proxy measure for educational status and an indication of military pensions being received. Higher ranks not included in this guidance (e.g. starred ranks) were incorporated into the senior officer category due to limited numbers and to ensure anonymity.

**Table 2.**

*Overview of rank categorisation for analysis*

| **Binary Variable** | **Categorical Variable** | **NATO Rank Code** | **Included ranks across all services** |
| --- | --- | --- | --- |
| Nonofficer | Junior Rank Rate | OR3 | Able Rate 1, Able Rate 2, Aircraftman, Marine, Private |
|  | Junior Non-Commissioned Officer | OR4 | Lance Corporal, Leading Aircraftman  Corporal, Senior Aircraftman, Leading Rating |
|  | Senior Non-commissioned Officer | OR6 to OR9 | Chief Technician, Petty Officer, Sergeant, Chief Petty Officer, Colour Sergeant, Staff Sergeant, Flight Sergeant, Warrant Officer 2, Command Sergeant Major, Staff Sergeant Major, Warrant Officer 1, Regimental Sergeant Major |
| Officer | Junior Officer | OF2 | Captain (army), Flight Lieutenant, Lieutenant, Sub-Lieutenant |
|  | Senior Officer | OF3 to OF6 and above | Lieutenant Commander, Major, Squadron Leader,  Commander, Lieutenant Colonel, Wing Commander,  Captain, Colonel, Group Captain, Commodore Brigadier, Air Commodore  Admiral, General, Air Marshal |
